# Development and Optimization of Dipyridamole- and Roflumilast-Loaded Nanoemulsion and Nanoemulgel for Enhanced Skin Permeation: Formulation, Characterization, and In Vitro Assessment

**DOI:** 10.3390/ph17060803

**Published:** 2024-06-19

**Authors:** Zeyad Khalaf Maded, Souad Sfar, Ghada Abd Alrhman Taqa, Mohamed Ali Lassoued, Olfa Ben Hadj Ayed, Hayder Adnan Fawzi

**Affiliations:** 1 Laboratory of Pharmaceutical, Chemical, and Pharmacological Drug Development LR12ES09, Faculty of Pharmacy, University of Monastir, Monastir 5000, Tunisia; representativekhd@gmail.com (Z.K.M.); lassoued98@yahoo.fr (M.A.L.); olfa.bha89@gmail.com (O.B.H.A.); 2 Laboratory of Chemical, Galenic and Pharmacological Development of Medicines (LR12ES09), Faculty of Pharmacy of Monastir, University of Monastir, Monastir 5000, Tunisia; souad.sfar@laposte.net; 3 Department of Dental Basic Sciences, College of Dentistry, University of Mosul, Mosul 41002, Iraq; ghadataqa@uomosul.edu.iq; 4 Department of Pharmacy, Al Mustafa University College, Baghdad 10064, Iraq

**Keywords:** psoriasis, nanoemulsion, dipyridamole, roflumilast, nanoemulgel, Franz cell diffusion

## Abstract

This study explores developing and optimizing a nanoemulsion (NE) system loaded with dipyridamole and roflumilast, aiming to improve skin penetration and retention. The NE formulation was further transformed into a nanoemulgel to enhance its application as a topical treatment for psoriasis. Solubility studies were conducted to select the oil, surfactant, and co-surfactant. Phase diagrams were constructed using the aqueous phase titration method. All the formulations were in nanoscale, and Formula (F2) (which contains oleic acid oil as the oil phase, a mixture of Surfactant Tween 80 and co-surfactant (ethanol) at a ratio of 1:2 in addition to distilled water as an aqueous phase in a ratio of 1:5:4, respectively) was the selected formula depending on the particle size, PDI, and zeta potential. Formula (F2) has the best ratio because it gives the smallest nanoemulsion globule size (particle size average of 167.1 nm), the best homogenicity (lowest PDI of 0.195), and the highest stability (higher zeta potential of −32.22). The selected formula was converted into a nanoemulgel by the addition of 0.5% (*w*/*w*) xanthan gum (average particle size of 172.7 nm) and the best homogenicity (lowest PDI of 0.121%) and highest stability (higher zeta potential of −28.31). In conclusion, the selected formula has accepted physical and chemical properties, which enhanced skin penetration.

## 1. Introduction

Psoriasis is a persistent skin condition caused by an overactive immune system, which leads to excessive growth of keratinocytes in the outer layer of the skin [1]. Nanoemulsions (NEs) are the most successful nano-based medications developed for treating psoriasis. Lipid-based nanoparticles boost skin penetration and reduce psoriasis symptoms by improving the delivery of anti-psoriatic medicines [2]. Based on the findings from research on irritation potential and in vivo assessments, it is evident that NEs can serve as an effective treatment for skin conditions such as atopic dermatitis and psoriasis [3]. The low viscosity of NE renders it inappropriate for use on human skin [4]; therefore, including a gelling agent with NE enhances the convenience of transdermal delivery [5]. Combining a gelling agent and nanoemulsion results in a dosage form known as nanoemulgel [6].

Roflumilast is a medication that inhibits phosphodiesterase type 4 (PDE4). It is prescribed for the long-term management of chronic obstructive pulmonary disease and asthma [7]. Roflumilast is an anti-inflammatory medication known to participate in multiple inflammatory processes. It reduces the levels of leptin, tumor necrosis factor (TNF)-α, interleukin (IL)-1b, IL-2, IL-13, interferon (IFN)-γ, and reactive oxygen species (ROS) [8,9,10]. The US Food and Drug Administration (FDA) has approved the use of roflumilast cream 0.3% (Zorvye; Arcutis Biotherapeutics) in the treatment of plaque psoriasis in both adolescents and adults [11]. This novel drug is the first topical PDE4 inhibitor approved for treating plaque psoriasis [12,13].

Dipyridamole (DIP) is a pharmaceutical drug that functions as an antiplatelet agent and inhibits the enzyme PDE3, enhancing intracellular cAMP/cGMP [14]. DIP, a coronary vasodilator, is often given orally and is now authorized for lowering the risk of thromboembolism after surgery [15,16]. In addition to the indirect anti-inflammatory actions of DIP through adenosine and prostaglandin I2, DIP can also directly reduce inflammation by inhibiting the interaction between platelets and monocytes [17]. In addition, DIP hinders the process of attracting, activating, and releasing proinflammatory substances by lymphocytes [18,19]. DIP was initially developed to suppress T cell-mediated immunoglobulin production by B lymphocytes [20]. The anti-inflammatory properties of DIP gel were demonstrated in a skin homogenate using a mouse model of imiquimod-induced psoriasiform skin inflammation [21]; other studies demonstrated antioxidant [22,23], neuroprotective [24], antiapoptotic [25], and antifibrotic effects [26] of dipyridamole in different tissues. In the current study, we hypothesize that combining both agents will act synergistically to enhance the biological activity of psoriasis due to similarities in their mechanisms of action; this is a novel combination that has never been reported in the literature.

In the last twenty years, medical nanotechnology advancements have led to numerous prospects and potential solutions for enhancing topical treatments for psoriasis [27]. NEs are colloidal carriers characterized by droplet sizes between 20 and 500 nm. NEs are created when two immiscible liquids, often water and oil, disperse together without forming visible phase boundaries due to their lack of solubility [28,29]. The system is appropriate for topical application because of its enhanced drug solubility, effective drug loading capacity, superior thermodynamic stability, and ability to enhance penetration without causing skin irritation [30,31]. NEs are isotropic systems that consist of lipids, surfactants, and co-surfactants. They can be either transparent or translucent when viewed optically. NEs can be classified into two categories: oil in water (O/W) or water in oil (W/O). The globules in NEs are often within the 5–500 nm size range [32,33]. Using NE as a carrier for anti-psoriatic medications is beneficial since it does not exhibit creaming, flocculation, sedimentation, or coalescence, typically found in macroemulsions [34]. NE consists of two main components: the oil phase base, composed of triacylglycerols, monoacylglycerols, diacylglycerols, and free fatty acids, and the incorporation of desired medicines and lipophilic surfactants. The aqueous phase mostly consists of water, surfactants, and co-surfactants [35].

Nanoemulgel is a promising method of delivering medications that aim to improve the effectiveness of lipophilic drugs. This innovative technique involves combining NE with a gel to boost its stability and enable the quick and controlled release of the drug [36,37]. The attention toward nanoemulgel has grown due to its capacity for precise administration, convenient application, lack of degradation in the gastrointestinal tract or initial metabolism, and favorable safety profile [36,38]. Nanoemulgel is a topically applied formulation that is based on emulsion. It involves the preparation of nanosized emulsion globules using either high-energy or low-energy processes. These globules are then transformed into nanoemulgel using an appropriate gelling agent [39]. A gelling agent is added to the NE preparation, and the two substances are combined to create a nanoemulgel. In this process, a liquid form of either water-in-oil (w/o) or oil-in-water (o/w) nanoemulsion is transformed into a dense and partially solid nanoemulgel using different polymeric gelling agents [40]. Various polymers, including Carbomer 940, Carbopol 943, Chitosan, Carbopol 934, Carbopol 940, Poloxamer 407, Methylcellulose, and xanthan gum, have been employed as gelling agents [41].

The nanoemulsion formulation was converted into a gel by incorporating three distinct concentrations of gel, specifically 0.5%, 1%, and 1.5%, to generate various formulations. Therefore, the uniqueness of this system resides in the fact that the constituents (oil, surfactant, and particularly co-surfactant) of the nanoemulgel functioned as agents that increased penetration [42]. Nanolipoidal formulations can create a nanoemulsion-based gel, also known as nanoemulgel, which is effective for delivering drugs through topical routes [43].

The current study aimed to develop, optimize, and physiochemically characterize a nanoemulsion system loaded with the proposed drug molecules (dipyridamole and roflumilast). It also involved preparing a nanoemulgel from the optimized NE formulation and assessing the in vitro skin permeation using appropriate methods, such as the Franz cell apparatus. 

## 2. Results and Discussion

### 2.1. Dipyridamole Solubility in Oils, Surfactants, and Co-Surfactants

**Figure 1:**
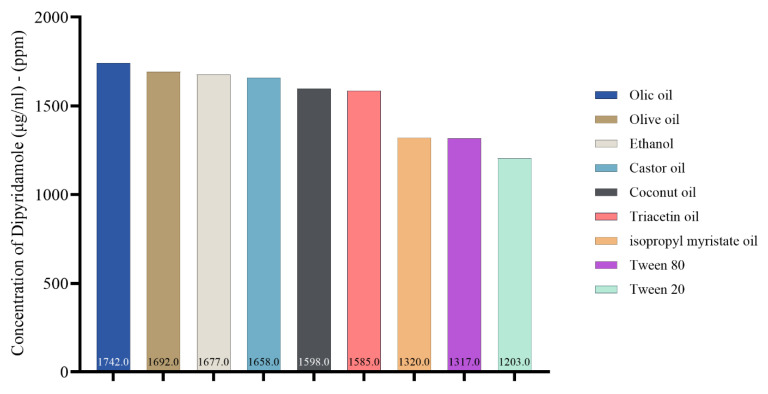
Concentration of dipyridamole (μg/mL), (ppm) in oils, surfactant, and co-surfactant.

Choosing a suitable oil for the oil phase is a crucial aspect of nanoemulsion formulation. The current study depicted the solubility of dipyridamole in various oils, surfactants, and co-surfactants for nanoemulsion, as shown in Figure 1. The solubilizing activity of oleic acid toward dipyridamole was found to be higher compared to other oils. Specifically, the solubilizing activity of oleic acid was measured at 1742 μg/mL, followed by olive oil at 1692 μg/mL, castor oil at 1658 μg/mL, and other oils. Figure S1 illustrates the HPLC analysis of dipyridamole in different oils, surfactants, and co-surfactants.

### 2.2. Roflumilast Solubility in Oils, Surfactants, and Co-Surfactants

**Figure 2:**
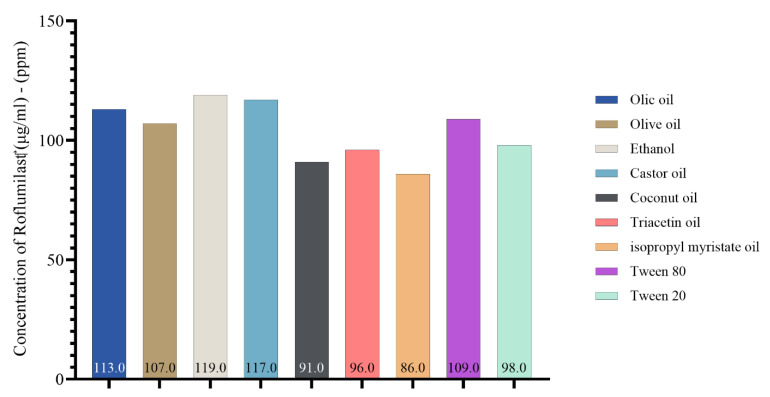
Concentration of roflumilast (μg/mL)–(ppm) in oils, surfactant, and co-surfactant.

In the present study, the solubility of roflumilast in different oils, surfactants, and co-surfactants for nanoemulsion is illustrated in Figure 2. Castor oil was found to have higher solubilizing activity toward roflumilast (μg/mL). It was found to be 117 μg/mL, followed by oleic acid at 113 μg/mL and olive oil at 107 μg/mL and then the other oils. Figure S2 illustrates the HPLC analysis of roflumilast in different oils, surfactants, and co-surfactants.

**Figure 3:**
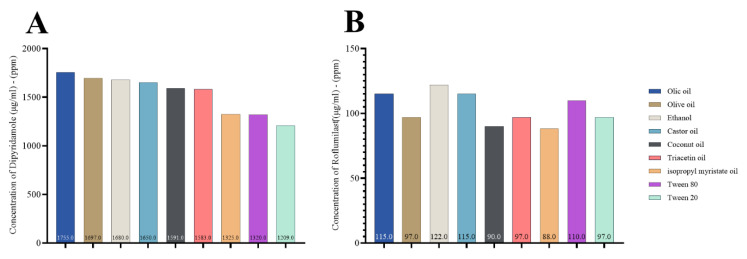
Concentration of dipyridamole and roflumilast combination (μg/mL)–(ppm) in oils, surfactant, and co-surfactant; (**A**) dipyridamole and (**B**) roflumilast.

Finally, we examined the solubility of both drugs in various oils, similar to that seen in the individual drugs. This indicates that the drugs are highly compatible when mixed and introduced to various oils, surfactants, and co-surfactants for nanoemulsion, as seen in Figure 3.

Compatibility testing is an essential stage in the formulation design process. Some oils/surfactants with high solubility did not effectively emulsify the medication. Compatibility tests can determine the emulsifying ability of different mixes, including various oils and surfactants; this allows for optimizing the combination of oil and surfactant [44].

According to Figure 2, roflumilast had greater castor oil solubility than other oils. However, it exhibited weak emulsifying capacity with the surfactants tested and was subsequently excluded. When selecting oleic acid as the oil phase, it was shown that Tween 80 was more effective in emulsifying than the other surfactants [45,46]. In the current study, we selected Tween 80 as a surfactant for the formulation of excipients. This choice was based on the previous findings that showed a greater solubilizing activity toward roflumilast and dipyridamole. Furthermore, Tween 80 exhibited greater solubility for dipyridamole, with a measured value of 1317 μg/mL, compared to Tween 20, which had a 1203 μg/mL solubility. Similarly, Tween 80 had stronger solubilizing activity for roflumilast, with a 109 μg/mL solubility, while Tween 20 had a lower solubility of 98 μg/mL.

Furthermore, ethanol exhibits a significant affinity for dipyridamole, with a 1677 μg/mL solubility. Additionally, ethanol demonstrates superior solubilizing properties for roflumilast, with a 119 μg/mL solubility. Azeem et al. and Park et al. demonstrated that selecting the primary components (co-surfactant, surfactant, and oil) during nanoemulsion preparation is a crucial factor in formulating development; this is because the drug’s solubility significantly influences the nanoemulsion’s capacity to keep the drug in a dissolved state in the oil phase [47,48]. 

### 2.3. Compatibility Study of Dipyridamole and Roflumilast and Drug-Excipients Mixtures Using FTIR Analysis

#### 2.3.1. FTIR Spectra Dipyridamole

In the present study, the typical peaks in the FTIR spectra of pure dipyridamole powder are 3751.55 cm^−1^ and 3381.21 cm^−1^ due to vibrational (N-H) stretching, 2922.16 cm^−1^ and 2848.86 cm^−1^ related to (=C-H) stretching, and 2333.87 cm^−1^ due to aliphatic (C-H) stretching. The aromatic C=C stretching is responsible for 1533.41 cm^−1^ and 1357.89 cm^−1^, aliphatic CH bending is responsible for 1282.66 cm^−1^ and 1215.15 cm^−1^, disubstituted orthobenzene stretching is responsible for 850.61 cm^−1^ to 665.44 cm^−1^, and CN stretching is responsible for 1078.21 cm^−1^ and 1018.41 cm^−1^. Figure S3 shows the FTIR spectra of the dipyridamole and its oils, surfactant, and co-surfactant.

#### 2.3.2. FTIR Spectra Roflumilast

In the present study, the typical peaks in the FTIR spectra of pure roflumilast powder are 3863.42 cm^−1^ to 3381.21 cm^−1^ due to vibrational (N-H) stretching, 2926.01 cm^−1^ related to (=C-H) stretching, and 2372.44 cm^−1^ due to aliphatic (C-H) stretching. The aromatic C=C stretching is responsible for 1656.85 cm^−1^ to 1386.82 cm^−1^, aliphatic CH bending is responsible for 1261.45 cm^−1^ and 1201.65 cm^−1^, disubstituted orthobenzene stretching is responsible for 873.75 cm^−1^ to 771.53 cm^−1^, and CN stretching is responsible for 1089.78 cm^−1^ and 873.75 cm^−1^. Figure S4 shows the FTIR spectra of the roflumilast.

#### 2.3.3. Drug and Excipient FTIR

The current work utilized FTIR to conduct a compatibility analysis of drugs and excipients. The objective was to identify any complexation or interaction between dipyridamole, roflumilast, and the excipients employed in the nanoemulsion preparation [49].

**Figure 4:**
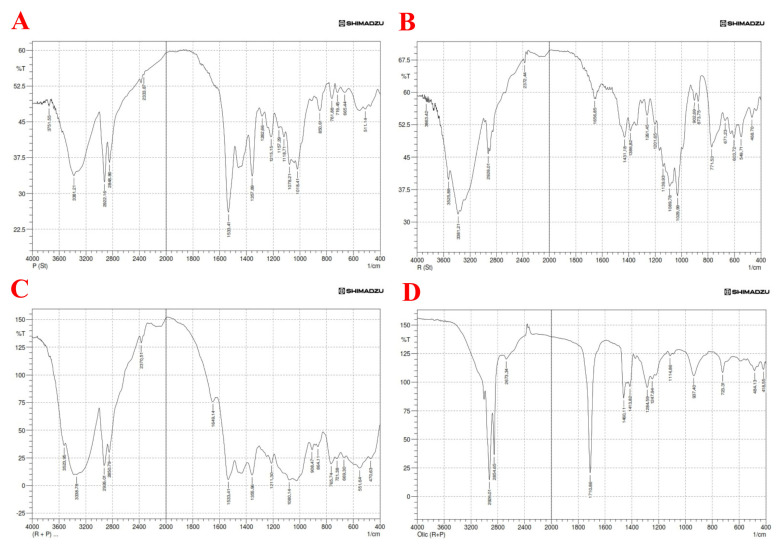
FTIR spectra of (**A**) pure dipyridamole, (**B**) pure roflumilast, (**C**) dipyridamole—roflumilast, (**D**) dipyridamole—roflumilast with oleic acid.

In the present study, analysis of the FTIR spectrum was performed to estimate both the drug purity (matching with reference peaks) and identify incompatibilities with fellow components of the selected formula. According to the findings obtained from FTIR results, there are no significant differences in the absorption of drug peaks among pure dipyridamole and roflumilast spectra. Therefore, it can be concluded that there is no chemical interaction or incompatibility between the drugs and other components used in the formulation, as illustrated in Figure 4 [50,51].

### 2.4. Pseudo-Ternary Phase Diagrams of Nanoemulsion

#### 2.4.1. Choose the Excipients for the Formulation (Oil (Oleic Acid), Surfactant (Tween 80), and Co-Surfactant (Ethanol)

The findings of the solubility study shed light on which components should proceed to the actual formulation step. Only oleic was selected for the oil phase, and the Tween 80 was considered part of the surfactant mixture (Smix) in various ratios with used co-solvents (ethanol) [52]. The surfactants chosen for o/w nanoemulsion preparation should have a hydrophilic–lipophilic balance (HLB) value between 12 and 18, a condition that both surfactants under study fulfilled [53]. Co-surfactants usually are short- or medium-chain alcohols with carbon atoms not more than eight atoms, and they allow a reduction in the amount required of surfactant and consequently lower safety-related issues since the biological toxicity of surfactants is usually higher than that of short-chain organic co-surfactants [53].

The surfactant Tween 80, which possesses high emulsifying activity and an HLB value of 15, was chosen. The presence of a significant or insignificant nanoemulsion area is determined by the ability of a specific surfactant or co-surfactant combination to dissolve the oil component. The degree of solubilization leads to an increased surface area with a more transparent and uniform solution. Observations revealed that using the surfactant Tween 80 in isolation did not significantly reduce interfacial tension and free energy when the surfactant ratio was increased from 1:1 to 3:1. Consequently, the development of nanoemulsion was limited to a smaller range [54]; see Table 1, Table 2 and Table 3.

**Figure 5:**
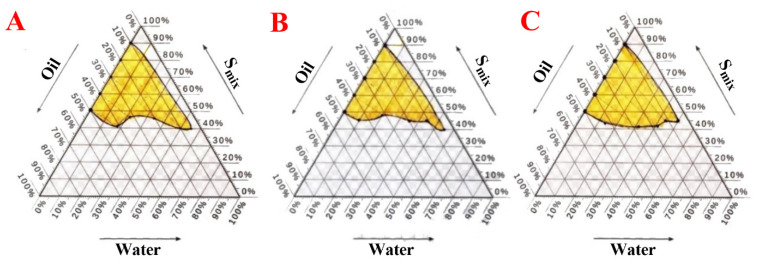
The pseudoternary phase diagrams depict the phase diagram for a mixture of oleic oil, Tween 80, and ethanol. (**A**) Formula (G1) with 1 (Tween 80):2 (ethanol) (Smix) and oleic oil and distilled water, (**B**) Formula (G2) with 1 (Tween 80):1 (ethanol) (Smix) and oleic oil and distilled water, (**C**) Formula (G3) with 2 (Tween 80):1 (ethanol) (Smix) and oleic oil and distilled water.

Ethanol is a low-molecular-weight co-surfactant that decreases the tension between two interfaces and enhances the movement of the hydrocarbon tail of the oil; this enables the oil to more easily enter the water phase [55]. In the present study, Formula G1 (Smix 1 (Tween 80):2 (ethanol) (Figure 5A), Formula G2 (Smix 1 (Tween 80):1 (ethanol) (Figure 5B), Formula G3 (Smix 2 (Tween 80):1 (ethanol) (Figure 5C).

#### 2.4.2. Pseudo-Ternary Phase for the Optimal Nanoemulsion Formula

**Figure 6:**
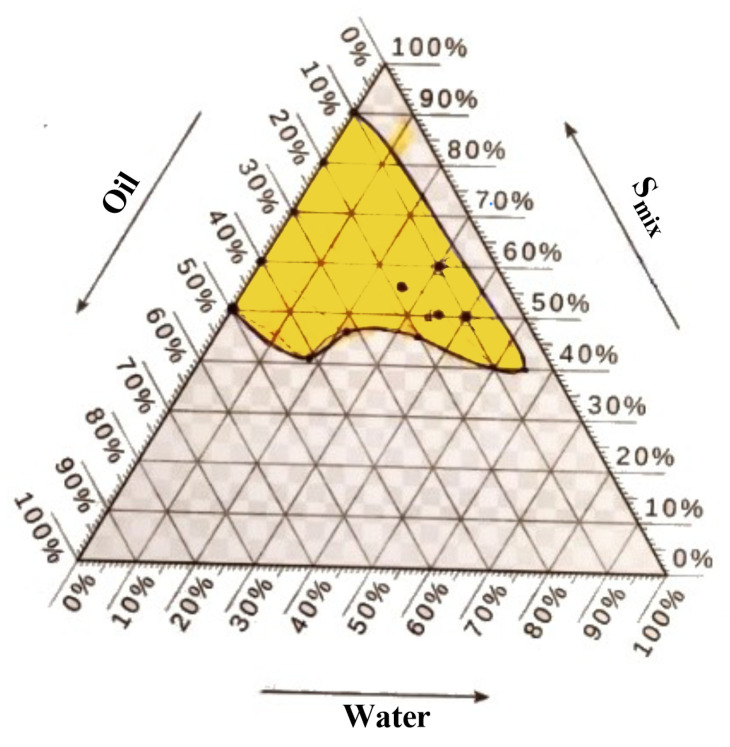
Phase diagram of Formula (F2) for a mixture of oleic oil, Tween 80, and ethanol (in a ratio of 1:2) along with distilled water.

Based on the acquired phase diagram area, the optimal formula for combining Smix and oil was chosen (G1). The medication was administered to oil and Smix and subjected to particle size analysis. A nanoemulsion was made by combining oleic acid as the oil phase with Tween 80 as the surfactant and ethanol as the co-surfactant in various ratios (1:1, 1:2, and 2:1), as indicated in Table 4. Formula (F2) was selected as the best formula, as illustrated in Figure 6.

### 2.5. Droplet Size and Polydispersity Index of Nanoemulsions

The chemical and physical features significantly influence the selection procedure. Several variables must be considered while creating a nanoemulsion tailored for a particular purpose. These parameters include the size of particles, the polydispersity index, the zeta potential, the pH, the viscosity, the stability, and the thermodynamic stability [56,57]. The dimensions and heterogeneity of nanoemulsions can impact characteristics such as particle stability, rheology, visual appearance, color, texture, and longevity. Ostwald ripening is nanoemulsions’ most common instability phenomenon [58,59].

#### 2.5.1. Particle Size

The mean droplet size of the investigated formulations ranged from 57.97 to 265.9 nm, as depicted in Figures S5–S8. The Formula (F2) chosen exhibits an approved size distribution due to its low polydispersible index, indicating a high level of quality and homogeneity (PDI 0.195). The item was inspected after a storage period of three months at three distinct temperatures. At temperatures of 4 °C, 25 °C, and 40 °C, there was no noticeable alteration in the results [60,61]. The polydispersity index quantifies the variability in particle sizes within a distribution. A monodisperse sample has polydispersity index (PDI) values close to zero. However, numbers ranging from 0.1 to 0.3 signify a narrow size range, values ranging from 0.1 to 0.4 signify a moderate size distribution, and values over 0.4 imply a broad size distribution. Most formulas complied with the PDI standards, except for Formulas F1 and F4, which unexpectedly deviated from the permitted range [62]. Among the evaluated formulas, Formula (F2) was chosen to be advanced for further testing due to it possessing the lowest PDI value. The droplets exhibit a high level of homogeneity due to the polydispersity index value being closer to zero. A higher polydispersity score suggests a lower level of homogeneity in the droplets of nanoemulsion compositions [63].

The solubility is increased because of the increase in surface area (decreasing particle size will lead to an increase in surface area), so the nanogel API will permeate easily compared to the API without nanogel formulation.

#### 2.5.2. Zeta Potential of Nanoemulsions

The zeta potential is a metric used to measure nanoemulsions’ surface charge characteristics and physical stability [64]. The zeta potential is a method used to analyze the surface charge of nanocrystals, providing valuable insights into the physical properties of a nanodispersion. A large positive or negative zeta value signifies the presence of electrostatic repulsion that prevents particles from coming close to each other. A low zeta value suggests that the particles will likely aggregate or flocculate due to Van der Waals interactions that bring the particles closer together. All findings, except for zeta values within the range of −30 mV to +30 mV, demonstrate that the dispersion exhibits sufficient repulsion among particles to establish a stable colloidal system [65].

#### 2.5.3. Suggested Nanoemulsion Formula 

Nanoemulsions were evaluated based on their average droplet size, polydispersity index, and zeta potential among the four formulations. All formulations exhibited comparable zeta potential values and demonstrated favorable particle size and distribution, as shown in Table 5. However, variations in droplet size, polydispersity index, and zeta potential resulted in the transformation of the F2 formulation into a nanoemulgel. Formula (F2) underwent gelation upon adding 0.5% *w*/*w* xanthan gum.

#### 2.5.4. Parameters Considered for Selection of Suggested Formula

The polydispersity index (PDI) assay measures the uniformity of droplet size in nanoemulsion formulations. The PDI has a numerical range of 0.0 to 1.0. When the polydispersity index value approaches zero, the droplets become more uniform in size and composition. A higher polydispersity score suggests a lower level of homogeneity in the droplets of nanoemulsion compositions [63].

In the prepared nanoemulsion formulas, the charge on an oil droplet is negative due to the presence of free fatty acids. The selected Formula (F2) exhibited a zeta potential of −32.22 mv, which indicates its stability. This information is found in Table 5. The product was examined during a storage period of three months under three distinct temperatures: 4 °C, 25 °C, and 40 °C. The findings indicated no substantial alteration [66,67].

The current study focuses on the selection of an optimum formula for nanoemulsions. The characterization study of the prepared nanoemulsions revealed that the optimized Formula (F2) exhibited desirable properties, including a small droplet size of 167.1 nm, a low PDI of 0.195, and a zeta potential of −32.22 mV. Further investigations, such as a stability study, will be conducted using the optimized formula. Oleic oil is regarded as the superior oil due to its ability to produce the smallest nanoemulsion globule size (with an average particle size of 167.1 nm), the best homogeneity (with a low PDI of 0.195), and the highest stability (with a higher zeta potential of −32.22 mV). A smaller droplet size enhances the surface area for drug transfer, release, and absorption, thereby improving bioavailability.

Furthermore, oleic oil exhibits greater solubility for dipyridamole, with a concentration of 1742 μg/mL, followed by olive oil at 1692 μg/mL and castor oil at 1658 μg/mL, surpassing the other oils. Furthermore, oleic oil exhibits a greater capacity for solubilizing roflumilast, with a measured value of 113 μg/mL, compared to olive oil, which measured 107 μg/mL—other oils showed even lower solubilizing activity [68].

Tween 80 is regarded as the most effective surfactant due to its capacity to produce the smallest nanoemulsion globule size (with an average particle size of 167.1 nm), the highest homogeneity (with the lowest PDI of 0.195), and the greatest stability (with a greater zeta potential of −32.22 mV). Reducing the size of the droplets enhances the overall surface area available for the medicine to be transferred, released, and absorbed, enhancing its bioavailability [33]. Furthermore, Tween 80 exhibits greater solubility toward dipyridamole, measuring 1317 μg/m, compared to Tween 20, which measures 1203 μg/m. Additionally, Tween 80 has stronger solubilizing activity toward roflumilast, measuring at 109 μg/m, whereas Tween 20 measures at 98 μg/mL [68].

In this study, various formulations of dipyridamole and roflumilast nanoemulsion were developed based on the different areas of the pseudoternary phase diagrams. The objective was to analyze these formulations and determine the optimal formula that exhibits the most desirable qualities. Formula (F2) is chosen due to its ability to produce smaller globule sizes, a low polydispersity index (PDI), and a greater zeta potential. The polydispersity index (PDI) is a metric used to evaluate the consistency of particle sizes in a nanosuspension, nanoemulsion, or nanogel. A higher polydispersity number suggests a lesser uniformity in the size of the globules in that preparation [69,70]. Co-surfactants, typically alcohols with chain lengths ranging from C3 to C8, are frequently included to decrease interfacial tension and enhance interface fluidity [71]. Additionally, they enhance the mobility of the hydrocarbon tail and facilitate deeper oil penetration into this area.

Alcohol can enhance the ability of the aqueous and oily phases to mix by distributing itself between these phases. Consequently, ethanol was chosen as a co-surfactant. Nanoemulsion systems are achieved by including co-surfactants at low concentrations of surfactants [72]. In this study, a mixture of Smix, with a ratio of 1 part Tween 80 to 2 parts ethanol, was prepared to decrease the amount of surfactant. Additionally, ethanol was found to have a high solubility of dipyridamole at a concentration of 1677 μg/mL and a higher solubilizing activity of roflumilast at a concentration of 119 μg/mL. A co-surfactant in nanoemulsion systems reduces the tension between the interfaces and enhances their ability to flow.

Furthermore, it enhances the mobility of the hydrocarbon tail and facilitates increased oil penetration within the nanoemulsion zone [73]. This study demonstrated that lipophilic substances, such as dipyridamole, are likely to experience notable changes in apparent solubility in the pre-prandial stage due to the influence of ethanol [74]. The nanoemulsion systems possess significant interfacial areas and stabilities, safeguarding molecules from unfavorable environmental conditions and enhancing their stability [75].

The phase diagrams of Formula (F2) demonstrate the greatest water retention capacity when the ratio of Tween 80 to ethanol is 1:2. Formula (F2) derived from the ratios above were chosen and prepared using dipyridamole and roflumilast. These formulas were then subjected to particle size, PDI, and zeta potential measurements. The formulation was created by dissolving 50 mg of dipyridamole and 15 mg of roflumilast in the specified amount of oleic acid. Four nanoemulsion formulations were produced in the current investigation, each containing varying proportions of the oily phase. The concentration of the surfactants was reduced to a minimum for the nanoemulsion. The formulation labeled F2 had the smallest globule size, measuring 167.1 nm, compared to all other formulations. The Formula (F2) formulation was chosen as the optimal formulation due to its compact size.

### 2.6. Thermodynamic Stability of Nanoemulsion

#### In Vitro Evaluation of Formulas (F1 to F4)

All four nanoemulsion formulas passed the three thermodynamic tests, demonstrating their thermodynamic stability. These nanoemulsions did not exhibit cracking, creaming, or phase separations, commonly observed in macroemulsions; this indicates that these preparations can withstand harsh and challenging storage conditions [48,76]. In the current investigation on thermodynamic stability, all formulated samples underwent a series of tests to assess their stability, including freeze–thaw cycling, heating/cooling cycling, and centrifugation. These tests were conducted to ensure the selection of a stable formulation, as indicated in Table 6 [77].

### 2.7. Nanoemulgel 

The gelling agent is a crucial constituent of nanoemulgel, providing consistency and smoothness to the formulation. These substances are cross-linking agents. Carbopol, Poloxamer, Tragacanth, HPMC, xanthan gum, and other similar substances are employed as gelling agents in the formulation of nanoemulgels [78]. Gel has greater viscosity than liquid nanoemulsion due to gel-forming polymers, such as carbopol or hydroxyl propyl methyl cellulose, which possess mucoadhesion properties [79]. The low viscosity of nanoemulsion renders it inappropriate for use on human skin. Utilize a gelling agent with nanoemulsion to enhance the ease of transdermal delivery [4,5].

### 2.8. Preparation of Nanoemulgel 

In this study, the selected formula was converted into a nanoemulgel by adding 0.5% (*w*/*w*) xanthan gum, then vertexing for a few minutes, and refrigeration. The resultant nanoemulgel was as clear as the original nanoemulgel but with a noticeable increase in viscosity. Higher concentrations of xanthan gum can also be used up to 1%, but obvious increases in viscosity will render administration more difficult and hinder drug release from its vehicle [80]. The suggested optimal formulation was converted into a nanoemulgel by the addition of 0.5% *w*/*w* of xanthan gum as a gelling agent (stirred with a vortex mixer for a few minutes, left for 1 h, then refrigerated overnight) that increases the consistency of your preparation and also possess mucoadhesive properties along with certain characteristics that confer it additional attributes for pharmaceutical formulation, like increasing the bioavailability of some poorly soluble drugs.

**Figure 7:**
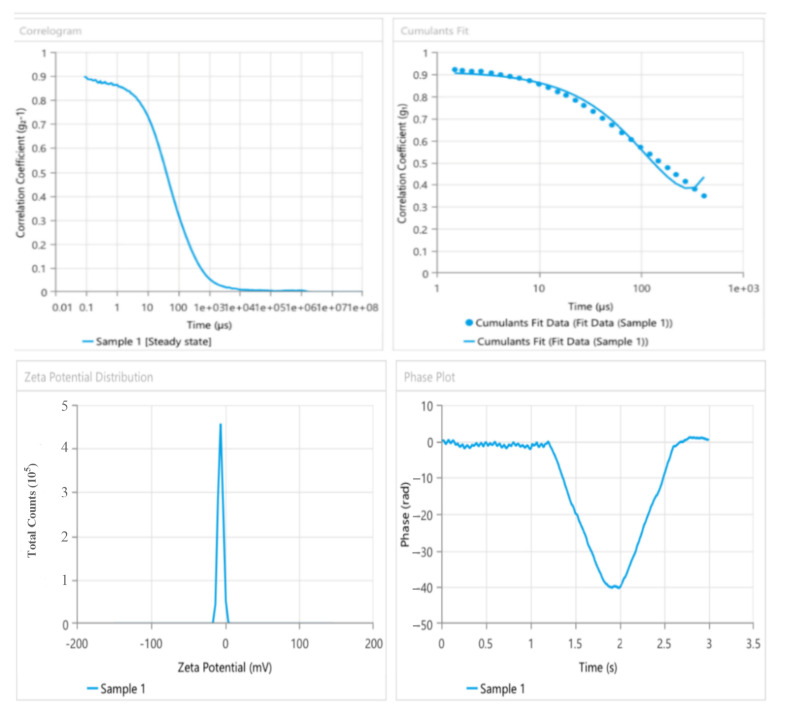
The particle size, PDI, and zeta potential of the nanoemulgel formula (phase diagram (1:2)).

As illustrated by Figure 7, the nanoemulgel formula had a particle size of 172.7 nm, 0.121% PDI, and a −28.31 mV zeta potential.

### 2.9. Viscosity of Nanoemulgel

The viscosity of nanoemulgel formulations was measured at various rotational speeds (10,12, 20, 30, 50, 60, 100, and 200 rpm) and a fixed temperature of 25 °C. Viscosity measurements are shown in Table S1.

The results of viscosity measurements of formulations were found to be in the range of 51 to 540 mPa/s for nanoemulgel, highlighting the significant boost in viscosity gained from xanthan gum addition to the formula, which is known for a dramatic hike in viscosity of such formulation upon its addition.

**Figure 8:**
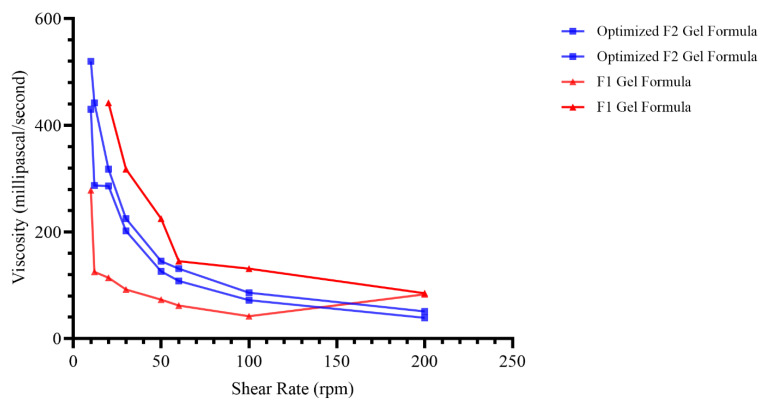
Viscosity values of formula at different shear stress (optimized formula versus F1 gel formula).

The results are shown graphically in Figure 8, showing the inverse relationship between the rotation speed (shear rate) and the reduced viscosity, indicating that the preparation flowed in a Newtonian [81,82]. The results of our study indicate that the enhanced formulation is appropriate for use on the skin and possesses satisfactory spreadability characteristics. A higher concentration of the gelling agent had a beneficial impact on the viscosity of the nanoemulgel formulation. As a result, the spreadability of the formulation is reduced since there is a reciprocal relationship between viscosity and spreadability [83,84].

### 2.10. Calibration Curve

See Supplementary File S1.

### 2.11. Ex Vivo Permeability Study

**Figure 9:**
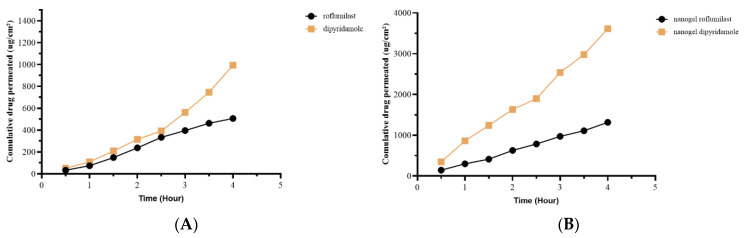
Permeability study of (**A**) pure roflumilast and dipyridamole and (**B**) nanogel roflumilast and dipyridamole.

Franz cell diffusion apparatus was used in this experiment to study the ex vivo permeation of the selected nanogel compared with pure roflumilast and dipyridamole gel [85]. The total quantity of roflumilast and dipyridamole nanogel that passed through the skin of rats is shown in Figure 9. The steady-state flow, permeability coefficient, and cumulative amount penetrated at 4 h from the nanogel were substantially more (*p*-value ≤ 0.05) than the plain roflumilast and dipyridamole pure gel. The diffusion parameters were also consolidated in Table 7.

So, the pure drug permeability was 35.3% for roflumilast and 20.8% for dipyridamole, while the Nanogel permeability was 91.8% for roflumilast and 75.6% for dipyridamole 75.6%.

## 3. Materials and Methods

### 3.1. Materials

Materials used in the current study include dipyridamole powder purchased from Hyperchem (Hangzhou, China), roflumilast purchased from Lupin Lab (Mumbai, India), ethanol (95%) from JT Baker (Shanghai, China), oleic acid from CDH (New Delhi, India), olive oil from Oilex S.A. (Madrid, Spain), coconut oil from Monastir (Tunisia), isopropyl myristate from CDH (India), caster oil from Monastir (Tunisia), triacetin oil from Monastir (Tunisia), Tween 80 (polyoxyethylene sorbitan monooleate) from HiMedia (Mumbai, India), Tween 20 (polysorbate 20) from HiMedia (India), xanthan gum from HiMedia Lab (India), formalin from Merck Chemicals (Darmstadt, Germany), and phosphate buffer saline from Titan Bio. Tech (Bhiwadi, India). 

The instruments used in the current study include an electric balance from Kern and Sohn GmbH (Balingen, Germany), an FTIR spectrophotometer, model 8300, from Lambda Scientific (Adelaide, Australia), a Franz diffusion cell (transdermal diffusion apparatus, Biobase, Jinan, China), high-speed centrifuges (15,000 rpm) from Hermle Z 216 MK (Wehingen, Germany), a hotplate with a magnetic stirrer (CB 162 Heat-Stir) by Stuart, Copley Scientific (Pocklington, UK), a particle size analyzer from Malvern Zetasizer (Worcestershire, UK), a sonicator from Copley Scientific (Nottingham, UK), a viscometer from Brookfield-DVE (Brookfield, WI, USA), a vortex mixer from Labinco L46 (Breda, The Netherland), a water bath with a shaker from G.F.L, Karl Kolb (Dreieich, Germany), a zeta potential analyzer from Malvern Zetasizer (UK), dissolution apparatus from RIGGETEK (Worcestershire, Germany), and an instrument for high-pressure liquid chromatography (HPLC) from Knauer (Berlin, Germany).

### 3.2. Methods

#### 3.2.1. Study Settings

The study was conducted between January 2022 and January 2024 in the Department of Pharmaceutics in the College of Pharmacy at Monastir University, Tunisia, and the animal study was conducted in the College of Pharmacy at Tikrit University, Saladin Province, Iraq.

#### 3.2.2. Determination of Dipyridamole and Roflumilast Solubility in Oils, Surfactants, and Co-Surfactants

A surplus amount of roflumilast and DIP, in a ratio of 6:94 mg/mg, respectively, was added to 5 mL of the excipient (oleic acid, Tween 80, ethanol, distilled water) in a glass tube with a screw lid. The solution was agitated at a temperature of 25 °C using an isothermal shaker for 48 to 72 h until it reached a state of equilibrium. Subsequently, each tube underwent centrifugation at a speed of 5000× *g* revolutions per minute for 15 min; we filtered the resultant solution (with a 0.45 μm filter syringe), and the supernatant was taken, which was mixed with methanol to create a diluted solution, and the concentration of the medication was measured using high-performance liquid chromatography (HPLC) [46,86,87].

##### HPLC for Dipyridamole

HPLC analysis was performed using isocratic elution on SYKAMN (Gewerbering, Germany), an HPLC system with column C18 − ODS (250 mm − 4.6 mm). The mobile phase is composed of a mixture of 0.1% orthophosphoric acid and acetonitrile in a ratio of 75:25 (*v*/*v*). The flow rate was 1.0 mL/min. Detection was performed at 227 nm; the injection volume was 100 µL [88,89].

##### HPLC for Roflumilast

The quantitative determination of roflumilast was performed using an HPLC method (SYKAM, Germany). The HPLC instrument used an isocratic method and a C18 − ODS column of 250 mm × 4.6 mm with a particle size of 5 μL. A 100 μL volume of roflumilast was used to inject the samples into the inject port. The data were evaluated using clarity software. The mobile phase was composed of a mixture of methanol and acetonitrile at a volumetric ratio of 25:75. The injections were performed at room temperature (30 °C) using a 100 μL loop, and the flow rate was set at 1.0 mL/min. The detection was carried out at a wavelength of 244 nm [90,91].

#### 3.2.3. Construction of Pseudo-Ternary Phase Diagrams

Pseudoternary schematics were created to analyze oil development in water nanoemulsion using four components: oil, surfactant, co-surfactant, and aqueous phase. The four-component system comprised (1) oleic acid as the oil phase, (2) Tween 80 as the surfactant, (3) ethanol as the co-surfactant, and (4) distilled water as the aqueous phase.

The pseudoternary phase diagrams were created by combining homogeneous liquid mixes of oil, surfactant, and co-surfactant with distilled water at ambient temperature (22.0 ± 2.0 °C). Oil, surfactant, and co-surfactant combinations were created in the necessary ratios of the surfactant and co-surfactant mixture (1:2, 1:1, and 2:1). Distilled water (DW) was incrementally added with gentle stirring to each liquid mixture according to the ratios specified in Table 1, Table 2 and Table 3. If turbidity was seen, followed by phase separation, the samples were classified as biphasic or monophasic if the mixes were clear and transparent after mixing. The samples were designated as points on the phase diagram. The points covered an area that corresponds to the location where nanoemulsion exists. The study reports all ratios as weight-to-weight ratios (*w*/*w*) [92,93,94].

#### 3.2.4. Preparation of Nanoemulsions

Nanoemulsions were synthesized using the aqueous phase titration technique. The nanoemulsion composition was selected based on the pseudoternary phase diagram (Figure 5). A solution was prepared by dissolving 50 mg of dipyridamole (1%) and 15 mg of roflumilast (0.3%) powder in the chosen oil. The selected surfactant and co-surfactant mixture were then added at the desired concentration. Water was gradually added with continuous stirring until a clear nanoemulsion was created [95,96].

Dipyridamole and roflumilast were precisely measured and dissolved in an appropriate amount of oil. Then, the specified amount of Smix was added to the drug mixture according to the formula. The mixture was vigorously mixed for 5 min at a speed of 100 using a vortexer. Finally, purified water was gradually added to obtain a clear oil-in-water nanoemulsion [21,97,98]. The higher concentrations of roflumilast cream (0.3%) were numerically better than those with the lower dose (0.15%) [99].

#### 3.2.5. In Vitro Evaluation of Formulas (F1 to F4), Visual Transparency

The optical transparency of the formulations was assessed by examining the sample in a transparent container under adequate lighting conditions while ensuring no reflection into the eyes. The sample was observed against a black and white lighted background [100,101].

##### Thermodynamic Stability Studies

Four formulations underwent three thermodynamic stability tests: centrifugation, heating/cooling, and freeze–thaw cycles. These tests evaluated their capacity to withstand various ambient conditions encountered during preparation and storage [100].

Centrifugation Test: The four formulations were centrifuged at 3500× *g* rpm for 30 min. During this process, the samples were carefully examined for signs of phase separation, creaming, coalescence, or cracking. The stable formulations were sent to the heating/cooling cycle [48,92].

Heating/cooling Cycle: The stability of a nanoemulsion was assessed by subjecting it to numerous cycles of high and low temperatures and then monitoring any phase changes using the centrifugation test discussed above. The refrigerator temperature fluctuates between 4 °C and a high temperature of 45 °C, with each temperature maintained for 48 h. This cycle is repeated six times. The formulations that remain stable at these temperatures were subjected to the freeze–thaw cycle [102].

Freeze–thaw Cycle: All four formulae underwent three freeze–thaw cycles alternating between deep freezing and room temperature. Each temperature was maintained for at least 48 h during storage. Formulations that successfully survived these thermodynamic stress tests were subjected to size analysis studies [103].

##### Droplet Size, Polydispersity Index, and Zeta Potential Measurement

The mean droplet size, polydispersity index, and zeta potential of particles were measured using a dynamic light scattering approach in a Zetasizer (Malvern Ltd., Fareham, UK). This technique analyzes the fluctuations in light scattering caused by the Brownian motion of droplets in their vehicles. The Zetasizer can precisely measure size ranging from 0.3 nm to 10 μm [104,105].

The droplet size was measured using photon correlation spectroscopy, a technique that examines the variations in light scattering caused by the random movement of the particles, known as Brownian motion. The device precisely measures dimensions within the range of 0.3 nm to 10 μm. Zeta potential quantifies the electric charge present on the surface of a nanoemulsion. Zeta potential was measured using specialized cuvettes. The polydispersity index (PDI) was calculated as the standard deviation ratio to the formulations’ mean droplet size. The PDI provides information on the size range of particles in the system. The polydispersity index measures the uniformity or consistency of the dispersion [61].

##### Drug and Excipient Compatibility Study by FTIR

The compatibility of the medication with the formulation was assessed using FTIR. The spectrum was acquired for the pharmaceuticals, oils, surfactants, and co-surfactants. The FTIR analysis combined the optimal formula, physical mixture, and drug individually with a small quantity of dry KBr powder. This mixture was then crushed onto a transparent disc, and the resulting spectra were recorded. These spectra were analyzed to verify the absence of any interaction between the medicine and the excipients, which might potentially occur throughout the procedure [106].

The sample is analyzed using Fourier Transform Infrared Spectroscopy (FTIR) at a modest scanning speed ranging from 4000 to 400 cm^−1^ to identify any interactions between the medicine and excipient [92]. FTIR analysis can be conducted to evaluate the interaction between drugs and excipients and examine polymerization and cross-linking processes. Additionally, it is employed to identify the functional groups together with their attachment method and the molecule’s unique characteristics [107].

#### 3.2.6. Preparation of Nanoemulgel

The suggested Formula (F2) was converted into a nanoemulgel by the addition of 0.5% *w*/*w* of xanthan gum as a gelling agent that also possesses mucoadhesive properties along with certain characteristics that confer additional attributes for pharmaceutical formulation like increasing the bioavailability of some poorly soluble drugs [108]. To accurately calculate the amount of xanthan gum required to achieve the 0.5% *w*/*w*, a sample of 5 mL of (F2) (50% Smix of 1:2 Tween 80:ethanol, 10% oleic acid, and 40% water; all in *v*/*v*%) was found to have 0.5% of xanthan gum, and 25 mg from the xanthan gum was added to 5 mL of the (F2) formula, stirred with a vortex mixer for few minutes, left for 1 h, and then refrigerated overnight [43,92].

#### 3.2.7. Viscosity Measurements of Nanoemulgel

The viscosity of the standard selected (F2) formula after adding the gelling agent was measured. The viscosity and rheological behavior of nanoemulgel were determined with a rotational digital viscometer with an R^2^ spindle [109]. Viscosity is a crucial element that reduces the fluidity and determines the required consistency of the nanoemulsion. Viscosity is crucial for maintaining the stability and controlling the release of drugs in nanoemulsion formulations. The viscosity of the emulsion is determined by the surfactant, water, and oil components, as well as their concentrations [110]. Five milliliters of (F2) was prepared, and xanthan gum 0.5% *w*/*w* was added and put in a glass beaker and adjusted to the recorded volume level (determined by the mark on the spindle shaft); measurements started at 37 ± 1 °C at six different speeds of 10, 12, 20, 30, 50, 60, 100, and 200 rpm with the corresponding reading recorded at each shear rate. Reverse reading from 200 to 10 rpm was also measured to evaluate any change that may occur during the recovery [85], and the rheological curve was obtained by plotting the recorded viscosity versus the spindle rotation rate.

#### 3.2.8. In Vivo Drug Delivery Study

##### Ex Vivo Permeability Study

Franz cell diffusion apparatus was used in this experiment to study ex vivo permeation [85] of the selected nanogel compared with pure roflumilast and dipyridamole gel. Five male Wister rats, each weighing approximately 200 ± 20 g, were used in the study. The Research Ethical Committee of the College of Pharmacy at Tikrit University, Saladin Province, Iraq, approved the animal study. All animals were provided with compassionate care, adhering to guidelines for the care and utilization of laboratory animals. Before the trial, the rats were allowed to consume only water without any food overnight. The rats were administered intraperitoneal anesthesia with a dosage of 80 mg/kg of ketamine and 10 mg/kg of xylazine. Following the administration of full anesthesia, the rats were killed by extracting blood through a puncture in the heart, which is a suitable procedure for collecting and preserving tissues [111,112]. After performing a 4–5 cm midline abdominal incision, the entire small intestine was separated, and an equal length of the skin segments was removed. The skin of the abdominal area that faces the intestine was obtained after the incision of the outer layer of the skin. The diameter of Franz cell diffusion was 2 cm, so we cut the skin into a circle with a diameter of 2 cm and a surface area of 3.14 cm^2^. The temperature was 37 °C, and the acceptor medium was phosphate buffer pH 7.4. The segments were promptly cleansed using an ice-cold normal saline solution administered through a syringe equipped with a blunt needle [113].

At an appointed time, a sample of 0.5 mL was taken and immediately replaced with an equal volume of new medium. The gathered samples were subjected to filtration and analysis to determine their levels of roflumilast and dipyridamole [114]. In this study, the cross-sectional area of the skin (S) was equal to 3.14 cm^2^, which was calculated by applying Equation (1) with a radius (r) of 1 cm.

(1) $$S=\frac{1}{2}r^{2}\pi$$

The apparent permeability coefficients (Papp) were calculated using Equation (2):

(2) $$P_{app}=\frac{\frac{dQ}{dt}}{\left(S\times C_{0}\right)}\;$$

where (dQ/dt)/S is the drug flux into the acceptor solution.

The steady-state rate (flux) can be determined by graphing the cumulative amount of drug permeated through the skin over time and analyzing the data using linear regression. The gradient of the linear segment of the graph would indicate the flux. Co denotes the initial drug concentration on the outer side. Permeation augmentation by formulation was achieved by comparing the steady-state permeation rate (flux) of selected nanogel formulae to the flux of pure roflumilast and dipyridamole gel. The total amount of roflumilast and dipyridamole that diffused into the acceptor jar after 4 h was also determined. The extension of the linear steady-state line to the time axis indicated the delay time.

## 4. Conclusions

A successful nanoemulsion (F2) preparation of dipyridamole and roflumilast has been achieved. The nanoemulsion composition includes dipyridamole and roflumilast at 1% and 0.3% *w*/*v*, respectively. The oil phase consists of oleic acid at a concentration of 10%. The surfactant and co-surfactant, Tween 80 and ethanol, are used in a 1:2 ratio at a concentration of 50%. Purified water makes up the remaining 40% of the composition. FTIR analysis confirms no interaction between the drug and the excipients used in the nanoemulsion preparation. The nanoemulgel that was prepared exhibits a greater and more rapid release of dipyridamole and roflumilast compared to pure dipyridamole and roflumilast; this results in enhanced drug absorption and improved bioavailability. The aqueous phase titration method is an effective technique for preparing drug nanoemulsions, and it is straightforward to use. Dipyridamole and roflumilast nanoemulsions were effectively formulated utilizing various oils and surfactants. Co-surfactant mixtures were prepared in various proportions using a pseudoternary phase diagram.

When choosing the oil, selecting the oil with the highest drug solubility is not always optimal. The selected formula, Formula (F2), consisted of oleic oil as the oil phase, Tween 80 as the surfactant, ethanol as the co-surfactant, and double-distilled water as the aqueous phase, in a ratio of 1:5:4, respectively. This formula exhibited a faster permeability rate compared to other formulas. Formula (F2) was chosen as the best option due to its small nanoemulsion globule size (average particle size of 167 nm), excellent homogeneity (PDI of 0.195), high stability (zeta potential of −32.9), and superior permeability rate compared to the others; to create a nanoemulgel, 0.5% (*w*/*w*) xanthan gum was added to the selected formula, resulting in an average particle size of 172.2 nm, improved homogeneity (PDI of 0.121), and increased stability (zeta potential of −28.31). The chosen formula demonstrates verified physical and chemical characteristics. Studies on the compatibility of drugs and excipients showed no chemical interaction between dipyridamole, roflumilast, and other components in the nanoemulsion preparation. The permeability coefficient and cumulative amount of substance that permeated after 4 h from the nanogel were notably higher than plain roflumilast and dipyridamole pure gel.

Based on the findings of the current study, we draw the following recommendations: evaluate the obtained formulation (nanoemulgel of dipyridamole and roflumilast) for in vivo anti-psoriatic potential by an imiquimod-induced psoriasis model in rats.

## Figures and Tables

**Table 1:** Formulations of nanoemulsions for pseudoternary diagrams and the percentage of the excipients for Formula G1 (Smix; 1 (Tween 80):2 (ethanol)).

	Oil (Tween 80):Smix (Ethanol) (2 mL)	Volume of Water to Turbidity	Total Volume
1	50 (1 mL):50 (1 mL)	0.4 mL	2.4 mL
2	40 (0.8 mL):60 (1.2 mL)	0.5 mL	2.5 mL
3	30 (0.6 mL):70 (1.4 mL)	1 mL	3 mL
4	20 (0.4 mL):80 (1.6 mL)	2 mL	4 mL
5	10 (0.2 mL):90 (1.8 mL)	2.5 mL	4.5 mL
Percentages of excipients			
	Oil%	Smix (%)	Distilled water
1	41.6	41.6	16.6
2	32	48	20
3	20	46.6	33.3
4	10	40	50
5	4.5	40	55.5

**Table 2:** Formulations of nanoemulsions for pseudoternary diagrams and the percentage of the excipients for Formula G2 (Smix; 1 (Tween 80):1 (ethanol)).

	Oil (Tween 80):Smix (Ethanol) (2 mL)	Volume of Water to Turbidity	Total Volume
1	50 (1 mL):50 (1 mL)	0.2 mL	2.2 mL
2	40 (0.8 mL):60 (1.2 mL)	0.5 mL	2.5 mL
3	30 (0.6 mL):70 (1.4 mL)	1.1 mL	3.1 mL
4	20 (0.4 mL):80 (1.6 mL)	1.5 mL	3.5 mL
5	10 (0.2 mL):90 (1.8 mL)	2.5 mL	4.5 mL
Percentages of excipients			
	Oil%	Smix (%)	Distilled water
1	45.5	45.5	9.1
2	32	48	20
3	19.4	45.2	35.5
4	11.4	45.7	42.8
5	4.4	40	55.5

**Table 3:** Formulations of nanoemulsions for pseudoternary diagrams and the percentage of the excipients for Formula G3 (Smix; 2 (Tween 80):1 (ethanol)).

	Oil (Tween 80):Smix (Ethanol) (2 mL)	Volume of Water to Turbidity	Total Volume
1	50 (1 mL):50 (1 mL)	0.3 mL	2.3 mL
2	40 (0.8 mL):60 (1.2 mL)	0.9 mL	2.9 mL
3	30 (0.6 mL):70 (1.4 mL)	1.3 mL	3.3 mL
4	20 (0.4 mL):80 (1.6 mL)	1.6 mL	3.6 mL
5	10 (0.2 mL):90 (1.8 mL)	2 mL	4 mL
Percentages of excipients			
	Oil%	Smix (%)	Distilled water
1	43.4	43.4	13
2	27.5	41.3	31
3	18.2	42.4	39.4
4	11	44.4	44.4
5	5	45	50

**Table 4:** Phase diagram 1:2 for Formula G1.

Formulation	Percentage			Volume (mL)		
	Distilled Water	Oil	Smix	Distilled Water	Oil	Smix
Formula (F1)	30	10	60	1.5	0.5	3
Formula (F2)	40	10	50	2	0.5	2.5
Formula (F3)	25	18	57	1.25	0.9	2.85
Formula (F4)	35	15	50	1.75	0.75	2.5
Total	100%			5 mL		

Note: to prepare 12 mL of Smix, a ratio of 4 mL of Tween 80 to 8 mL ethanol was used (1:2 ratio).

**Table 5:** Physical characteristics of various formulas.

Formula	Smix:Oil Ratio	Average Droplet Size (nm)	PDI (%)	Zeta Potential (mV)
F1	6:1	58	0.573	−6.08
F2	5:1	167	0.195	−32.22
F3	5.7:1.8	151	0.533	−18.68
F4	5:1.5	247	0.417	−21.23

**Table 6:** Thermodynamic stability of Formulas (F1–F4).

Formula Number	Centrifugation Test	Freeze–Thaw Cycles	Heating/Cooling Cycles
F1	pass	pass	pass
F2	pass	pass	pass
F3	pass	pass	pass
F4	pass	pass	pass

**Table 7:** The diffusion parameters.

Formula Code	Cumulative Amount Diffused at 4 h (Q4 h·µg)	Flux (dQ/dt·S) (µg/min·c^2^)	Permeability Coefficient (P_app_ × 10^−3^ cm/h)
Pure roflumilast	1589	2.9	58
Pure dipyridamole	3123	2.6	20.8
Roflumilast nanogel	4135	3.83	76.6
Dipyridamole nanogel	11,345	12.66	101

## Data Availability

Data is contained within the article or Supplementary Material.

## Supplementary Materials

The following supporting information can be downloaded at: https://www.mdpi.com/article/10.3390/ph17060803/s1, Table S1: Viscosity Nano-Emulgel (millipascal/second). Figure S1. HPLC analysis of (**A**) pure dipyridamole, (**B**) dipyridamole with oleic, (**C**) dipyridamole with olive oil, (**D**) dipyridamole with ethanol, (**E**) dipyridamole with castor oil, (**F**) dipyridamole with coconut oil, (**G**) dipyridamole with triacetin oil, (**H**) dipyridamole with tween 20, (**I**) dipyridamole with tween 80, and (**J**) dipyridamole with isopropyl myristate oil, Figure S2: HPLC analysis of (**A**) pure roflumilast, (**B**) roflumilast with oleic, (**C**) roflumilast with olive oil, (**D**) roflumilast with ethanol, (**E**) roflumilast with castor oil, (**F**) roflumilast with coconut oil, (**G**) roflumilast with triacetin oil, (**H**) roflumilast with tween 20, (**I**) roflumilast with tween 80, and (**J**) roflumilast with isopropyl myristate oil, Figure S3: FTIR spectra of (**A**) coconut, (**B**) ethanol, (**C**) isopropyl myristate, (**D**) olive oil, (**E**) oleic oil, (**F**) castor oil, (**G**) triacetin oil, (**H**) tween 20, (**I**) tween 80, (**J**) dipyridamole, (**K**) dipyridamole with ethanol, (**L**) dipyridamole with tween 80, (**M**) dipyridamole with oleic oil, Figure S4: FTIR spectra of (**A**) roflumilast, (**B**) roflumilast with ethanol, (**C**) roflumilast with tween 80, (**D**) roflumilast with oleic oil, (**E**) Dipyridamole – Roflumilast, (**F**) Dipyridamole/ Roflumilast with oleic acid, Figure S5: the particle size, PDI, and Zeta Potential of Formula F1, Figure S6: the particle size, PDI, and Zeta Potential of Formula F2, Figure S7: the particle size, PDI, and Zeta Potential of Formula F3, Figure S8: the particle size, PDI, and Zeta Potential of Formula F4. File S1. Calibration Curve. References [88,91,115] are cited in the supplementary materials.
