# Supplementary material for: Development and Optimization of Dipyridamole- and Roflumilast-Loaded Nanoemulsion and Nanoemulgel for Enhanced Skin Permeation: Formulation, Characterization, and In Vitro Assessment
Source: Pharmaceuticals (Basel). 2024 Jun 19;17(6):803. doi: 10.3390/ph17060803 (PMC11207013; doi:10.3390/ph17060803)
Supplement: Supplementary file 1 [file pharmaceuticals-17-00803-s001.zip › Supplementary File S1‎.pdf]

3.10 Calibration Curve

See Supplementary File S1.

3.10.1 Calibration Curve for Dipyridamole

An HPLC analysis was conducted utilizing isocratic elution using a SYKAMN - German HPLC system with a C18 - ODS column of 250 mm x 4.6 mm. The mobile phase consists of a blend of 0.1% orthophosphoric acid and acetonitrile at a volumetric ratio of 75:25. The flow rate was 1.0 milliliters per minute. Five standard test tubes were used to hold diverse amounts of a standard stock solution of dipyridamole with a concentration of 100ug/ml. The solution was then diluted to different concentrations ranging from 5-30 µg/ml, and each sample was injected. The measurements of the solutions were documented at a wavelength of 227 nm (Figure 1A) [1, 2].

3.10.2 Calibration Curve for Roflumilast

The quantitative determination of Roflumilast was performed using a high-pressure liquid chromatographic (HPLC) method with a German SYKAM apparatus. The HPLC instrument utilized was an isocratic system with a C18 - ODS column measuring 250 mm x 4.6 mm and having a particle size of 5µ. A 100µL volume of Roflumilast inject port was utilized to inject the samples. The data was evaluated using the clarity software. The mobile phase was composed of a mixture of Methanol and Acetonitrile at a volumetric ratio of 25:75. The injections were performed at room temperature (30°C) using a 100 µl loop, and the flow rate was set at 1.0 ml/min. Five standard test tubes were used in this experiment. Different amounts of a standard stock solution of roflumilast, with a concentration of 100ug/ml, were added to each test tube. The concentrations of roflumilast in the test tubes ranged from 5-30 µg/ml. Each sample was then injected. The measurements of the solutions were documented at a wavelength of 244 nm (Figure 1B) [3].

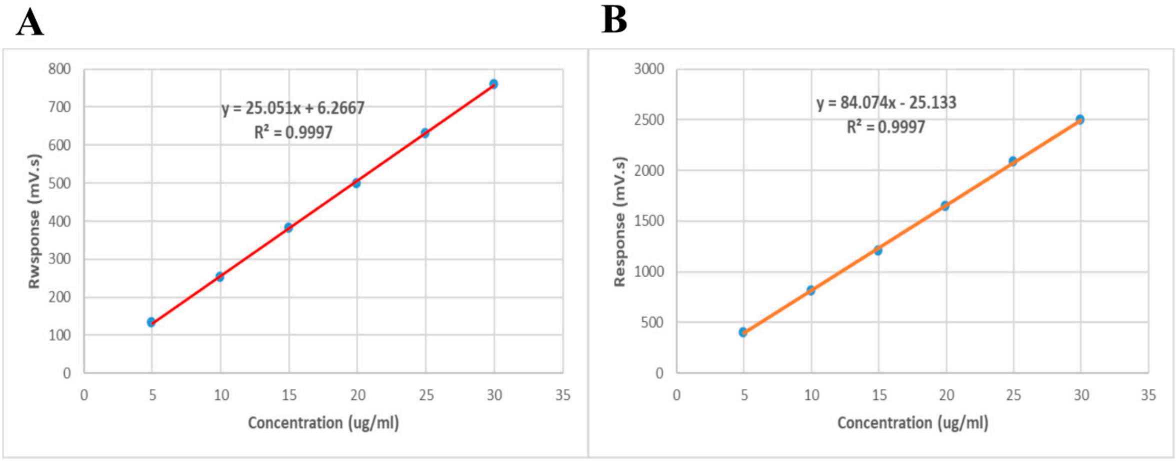

Figure 1: Calibration curve of (A) Dipyridamole in phosphate buffer pH 7.4 and (B) roflumilast in phosphate buffer pH 7.4

References

1. Prakash K, Kalakuntla RR, Sama JR. Rapid and simultaneous determination of aspirin and dipyridamole in pharmaceutical formulations by reversed-phase high performance liquid chromatography (RP-HPLC) method. African Journal of Pharmacy and Pharmacology. 2011;5(2):244-51,

2. Barghash SS, Abd El-Razeq S, El -Awady M, Belal F. Validated Spectrophotometric method for analysis of Dipyridamole and Lamivudine using eosin Y. Azhar International Journal of Pharmaceutical and Medical Sciences. 2021;1(1):87-96.DOI:10.21608/aijpms.2021.52761.1016
3. Belal TS, Ahmed HM, Mahrous MS, Daabees HG, Baker MM. Validated stability-indicating HPLC-DAD method for determination of the phosphodiesterase (PDE-4) inhibitor roflumilast. Bulletin of Faculty of Pharmacy, Cairo University. 2014;52(1):79-89.DOI:https://doi.org/10.1016/j.bfopcu.2014.04.002
