# Supplementary material for: Development and Optimization of Dipyridamole- and Roflumilast-Loaded Nanoemulsion and Nanoemulgel for Enhanced Skin Permeation: Formulation, Characterization, and In Vitro Assessment
Source: Pharmaceuticals (Basel). 2024 Jun 19;17(6):803. doi: 10.3390/ph17060803 (PMC11207013; doi:10.3390/ph17060803)
Supplement: Supplementary file 1 [file pharmaceuticals-17-00803-s001.zip › Table S1.pdf]

Table S1: Viscosity Nano-Emulgel (millipascal/second)

rpm	Viscosity Nanoemulgel	rpm	Viscosity Nanoemulgel
**10**	540	**100**	85
**12**	458	**60**	132
**20**	327	**50**	153
**30**	237	**30**	235
**50**	153	**20**	334
**60**	131	**12**	520
**100**	86	**10**	NA
**200**	51		
