# Supplementary figures and images for: Development and Optimization of Dipyridamole- and Roflumilast-Loaded Nanoemulsion and Nanoemulgel for Enhanced Skin Permeation: Formulation, Characterization, and In Vitro Assessment
Source: Pharmaceuticals (Basel). 2024 Jun 19;17(6):803. doi: 10.3390/ph17060803 (PMC11207013; doi:10.3390/ph17060803)

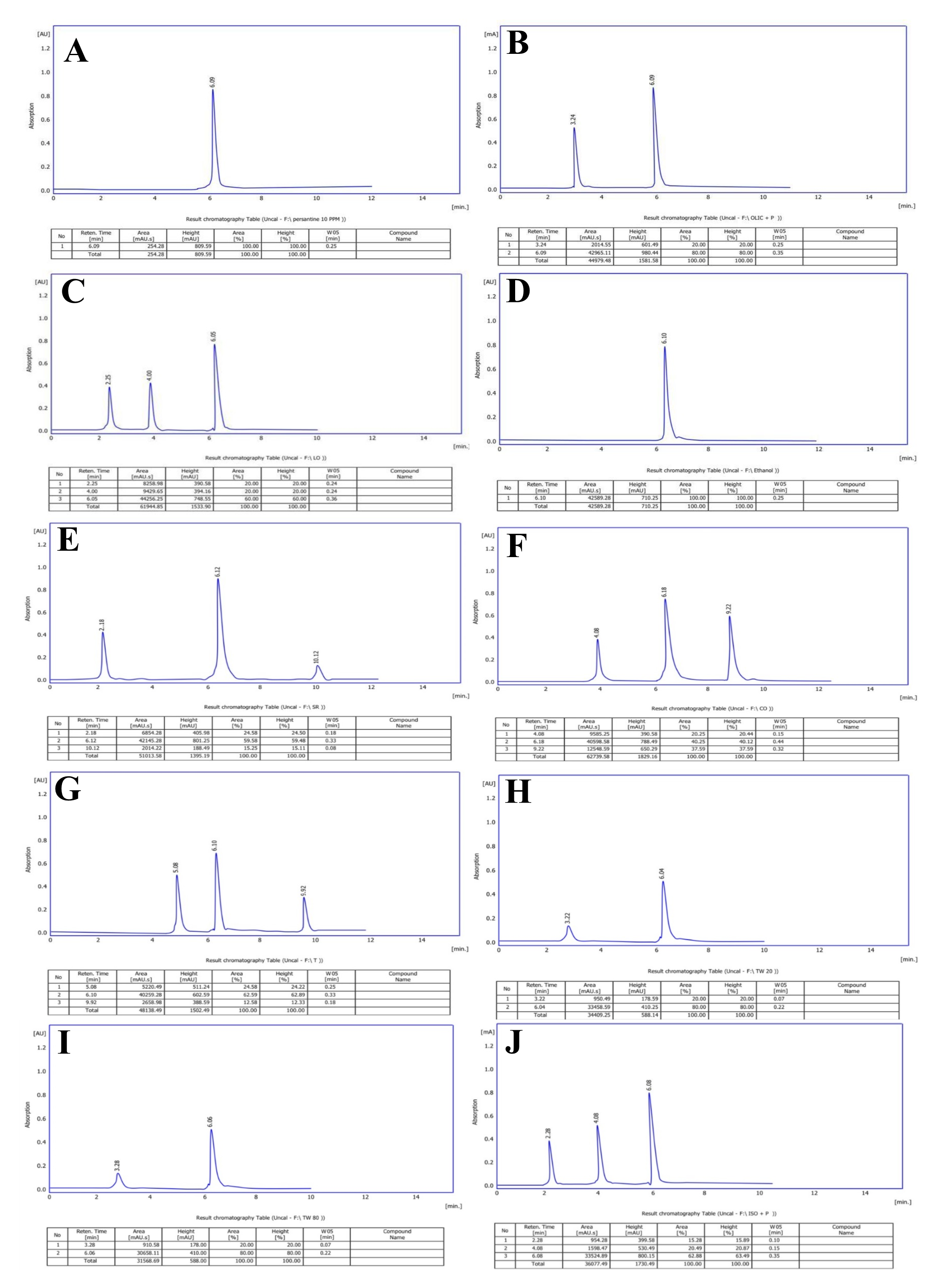

Supplement: Supplementary file 1 [file pharmaceuticals-17-00803-s001.zip › Figure S1.tif]

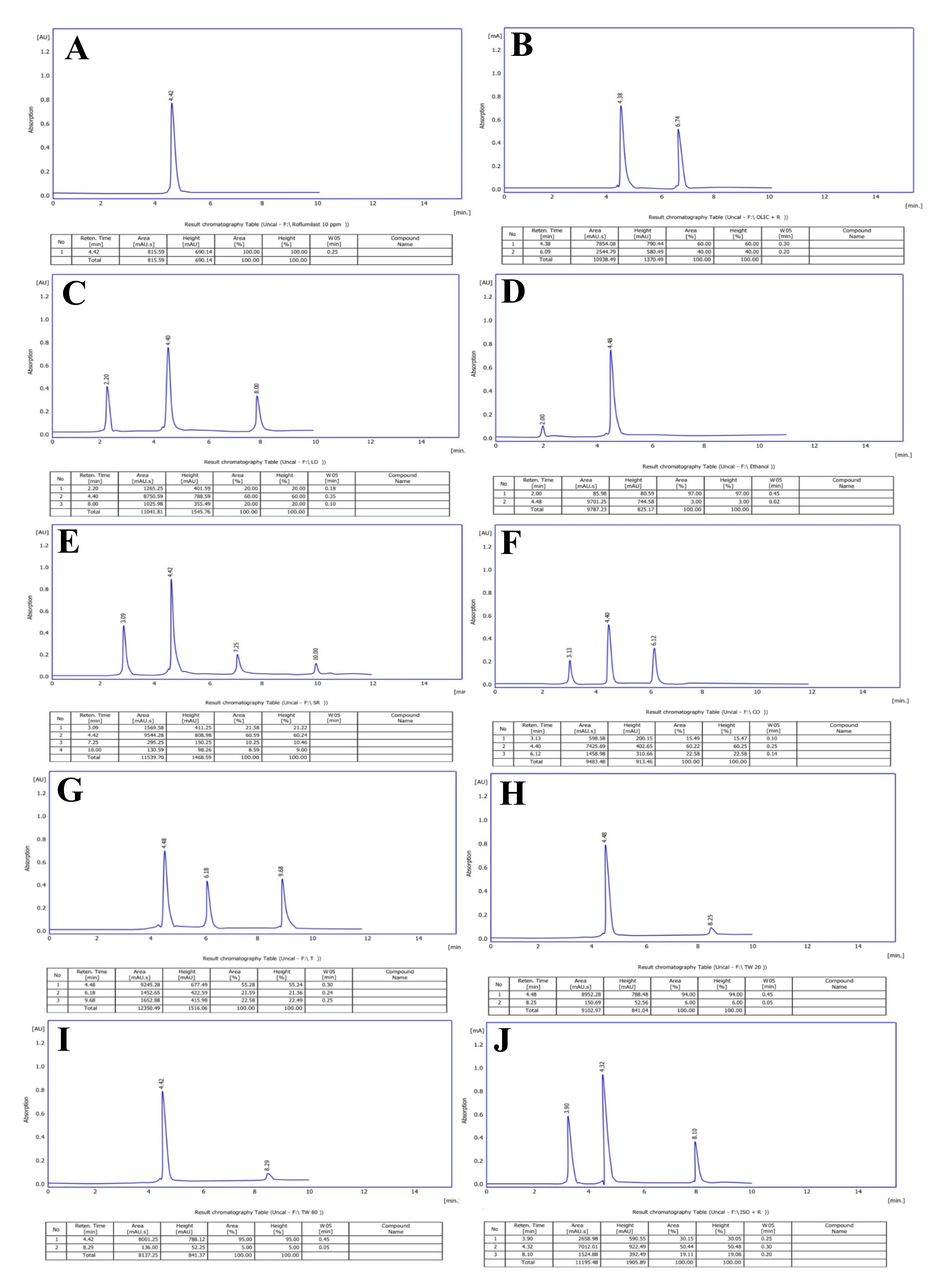

Supplement: Supplementary file 1 [file pharmaceuticals-17-00803-s001.zip › Figure S2.tif]

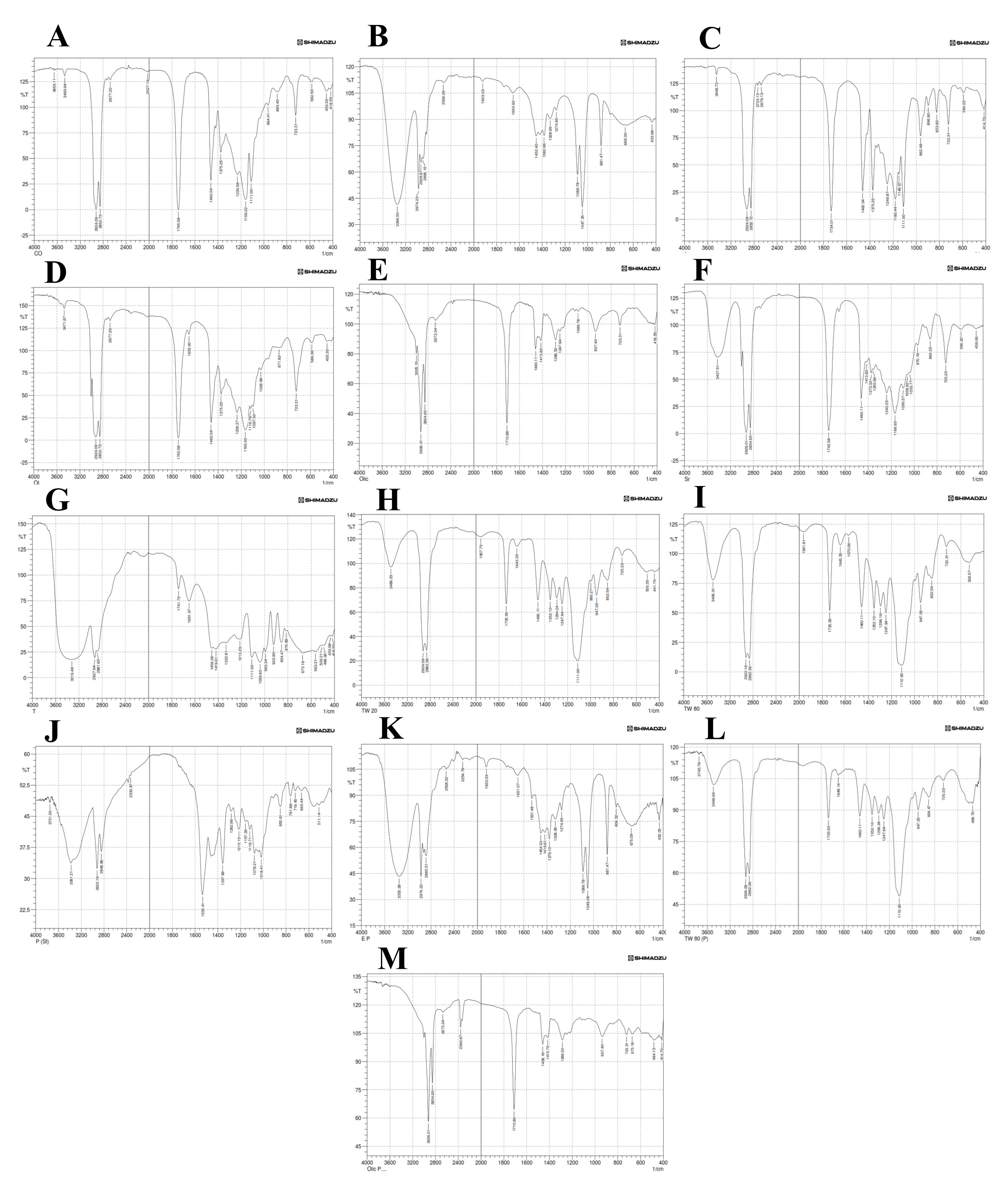

Supplement: Supplementary file 1 [file pharmaceuticals-17-00803-s001.zip › Figure S3.tif]

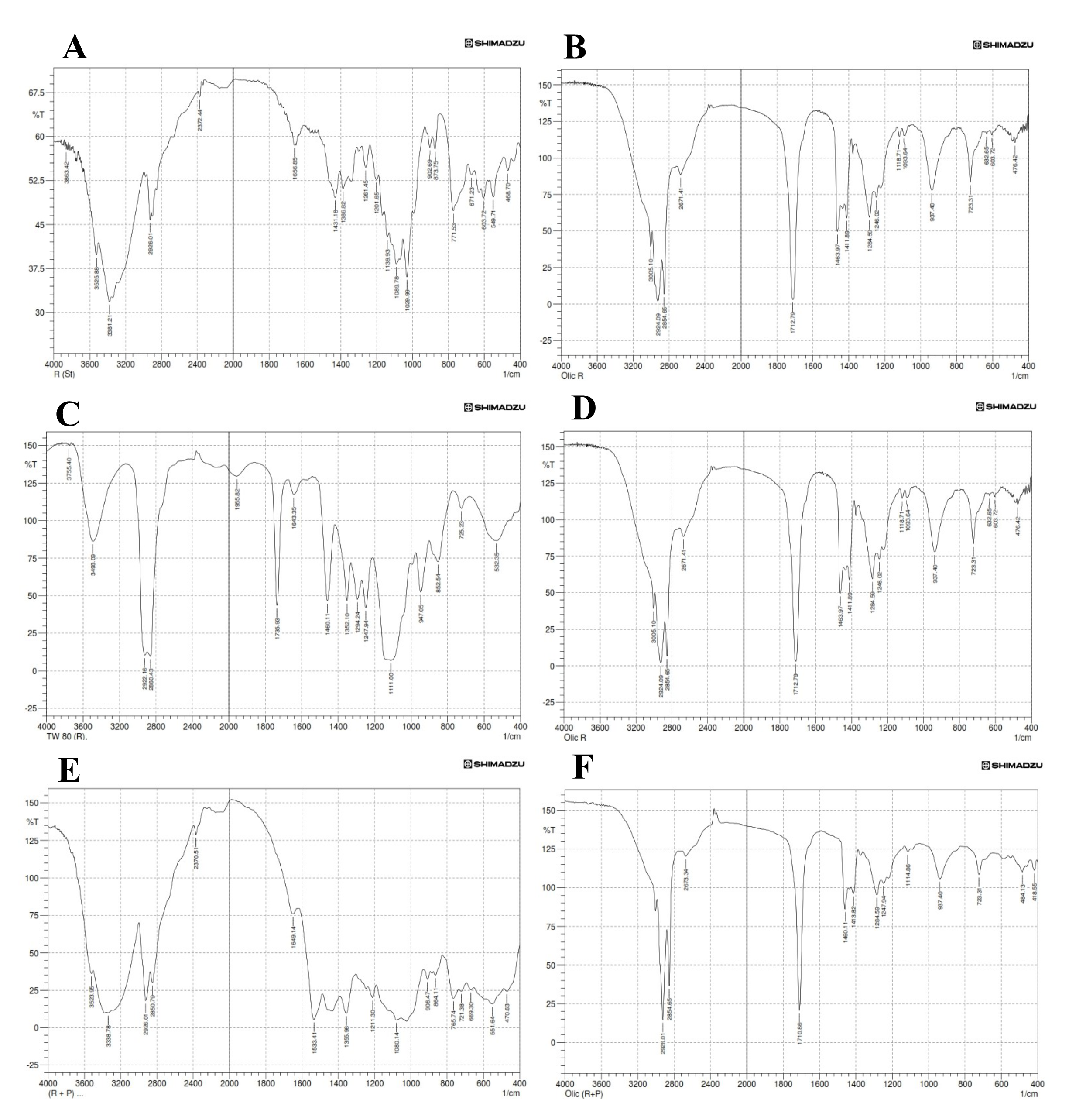

Supplement: Supplementary file 1 [file pharmaceuticals-17-00803-s001.zip › Figure S4.tif]

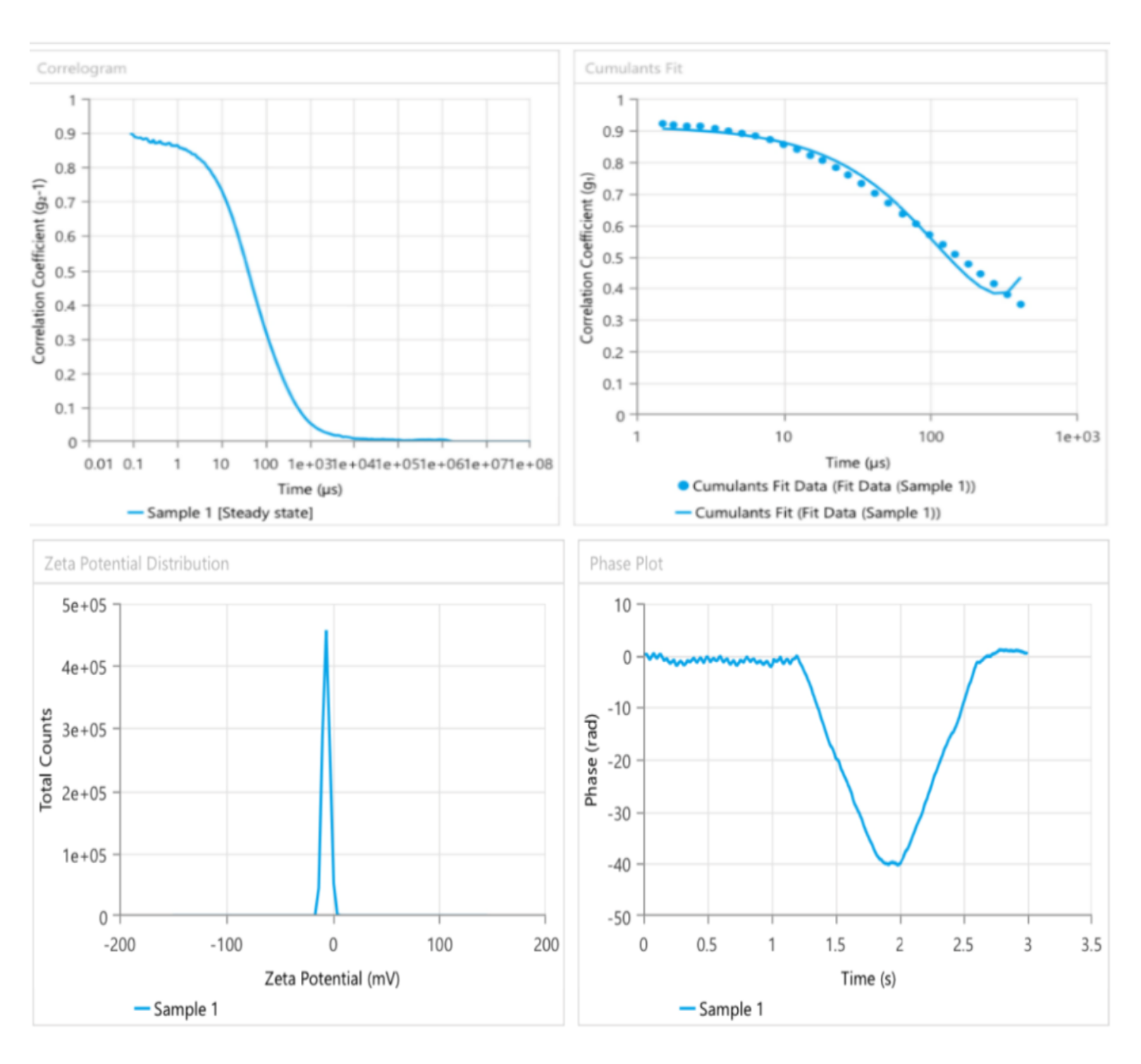

Supplement: Supplementary file 1 [file pharmaceuticals-17-00803-s001.zip › Figure S5.tif]

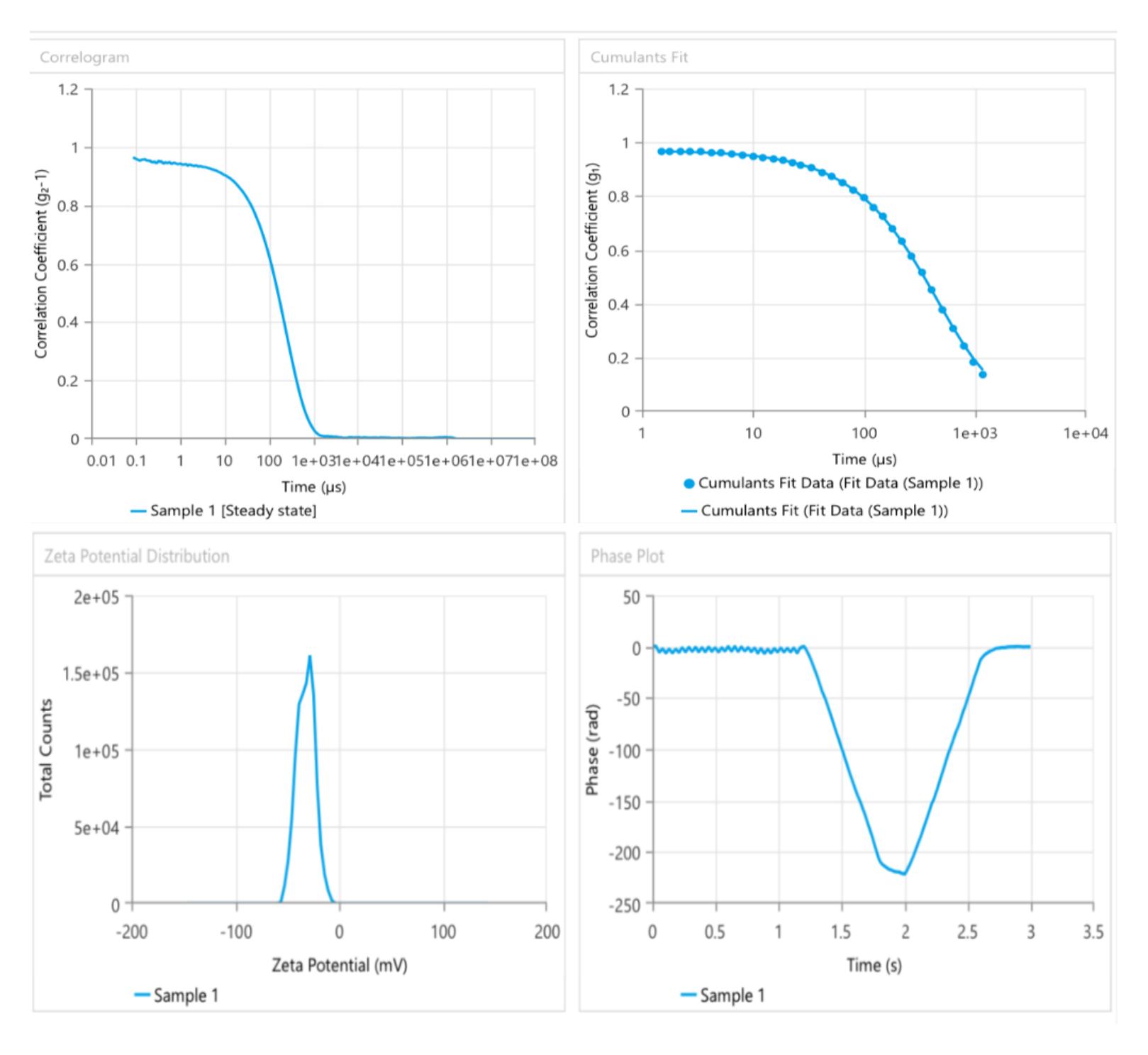

Supplement: Supplementary file 1 [file pharmaceuticals-17-00803-s001.zip › Figure S6.tif]

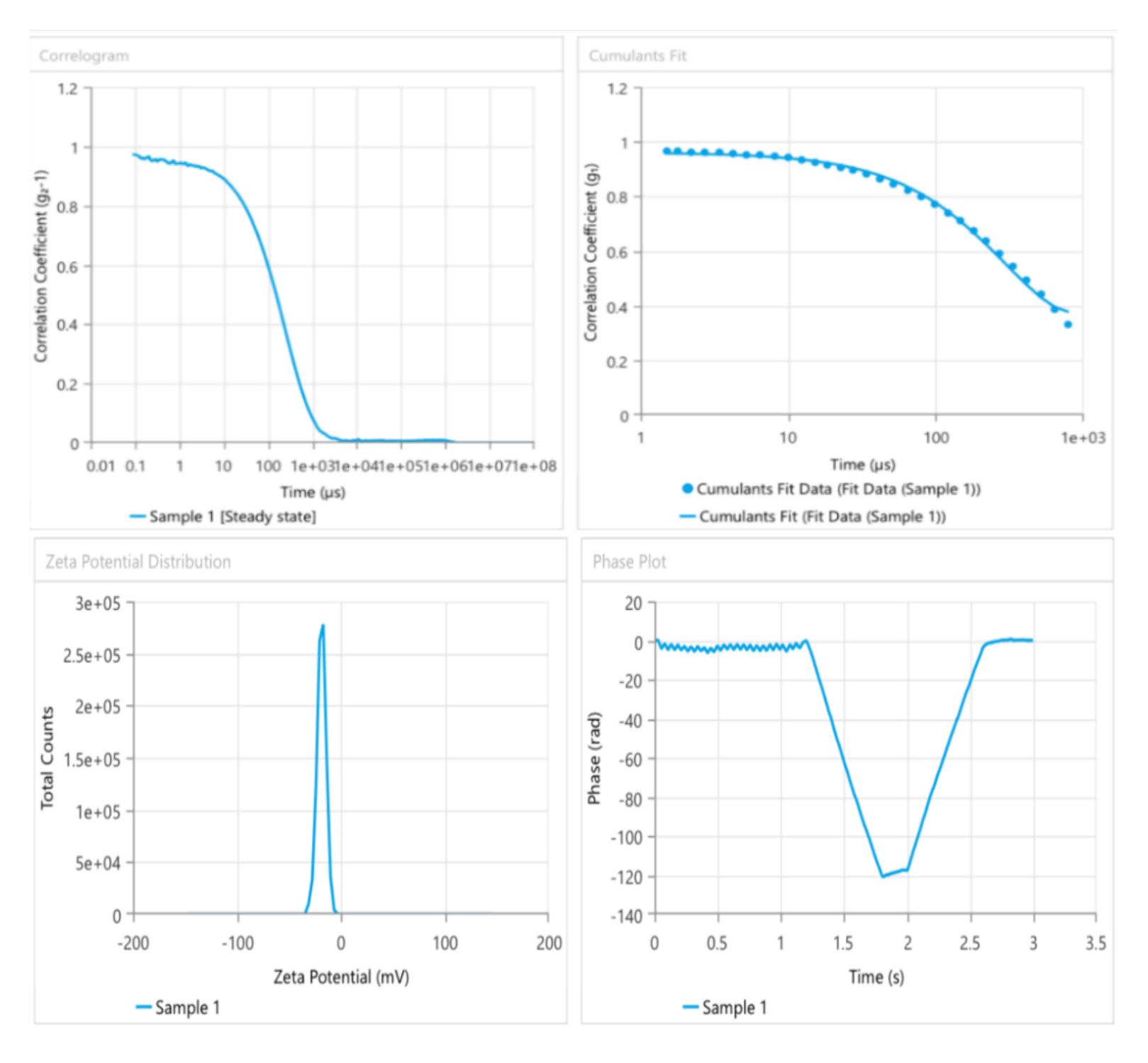

Supplement: Supplementary file 1 [file pharmaceuticals-17-00803-s001.zip › Figure S7.tif]

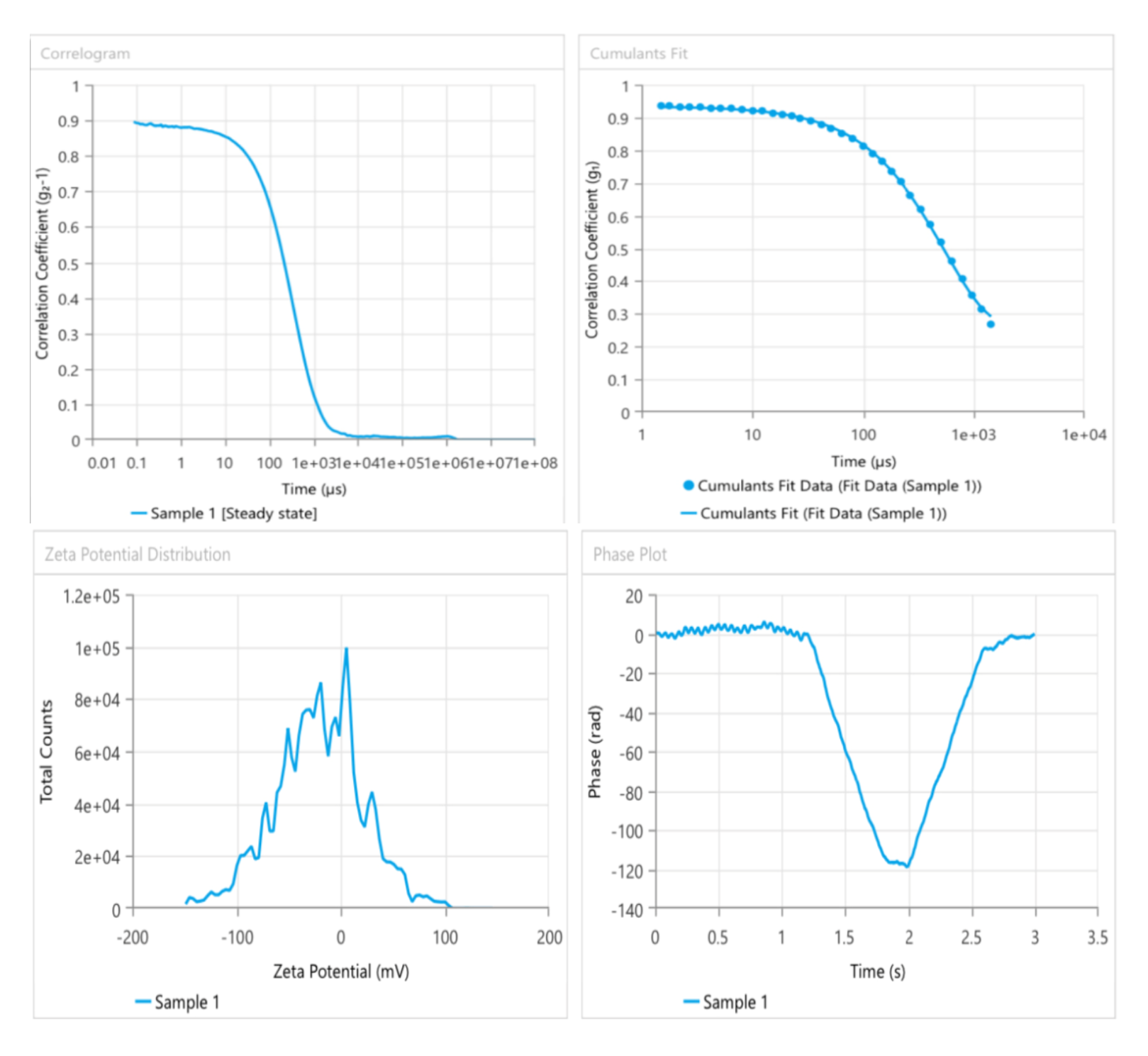

Supplement: Supplementary file 1 [file pharmaceuticals-17-00803-s001.zip › Figure S8.tif]
